# “IgG’s: contending with aggregating circumstances”

**DOI:** 10.15406/ijmboa.2024.07.00163

**Published:** 2024-03-12

**Authors:** Paul Toran, Lisa Arvidson, Alexandra Vachon, Don M Wojchowski

**Affiliations:** University of New Hampshire, Molecular, Cellular and Biomedical Sciences, USA

## Opinion

IgG antibodies are in increasing demand for use as therapeutic humanized monoclonal antibodies (mAb’s),^1,2^ mass- tagged antibodies for spatial imaging of sectioned tissue biopsies^3,4^ and quantitative assays of disease markers and pathogens. During the production of such antibodies (typically as mAb’s), compromising aggregation events can be encountered during producer cell culture, antibody purification, storage and/or covalent labeling.^5–8^ Native structures and conformations of IgG’s achieve a minimal free-energy state dictated by composite electrostatic interactions, hydrogen bonds, Van der Waals effects, side-chain flexibility, and hydrophobic effects.^8^ As IgG monomer concentrations increase, inter-molecular interactions can occur to the extent of forming aggregated monomers. Here, unfolded states can expose hydrophobic cores as prime drivers of folding events.^8^ Unfolded states can also be triggered (and propagated) by solvents, and by the covalent labeling of antibodies. Needs therefore exist for not only approaches that limit IgG aggregation, but also for the informative assay of IgG aggregates.

The continued need to address IgG aggregation is underscored first by recent examples of approaches aimed at limiting aggregate formation. These include new surfactants for producer cell cultures such as Peptronic (a potential substitute for Pluronics);^9^ Protein A resins that (during the acidified elution of isolated antibodies) release isolated IgG’s at higher pH (e.g, pH 4.5);^10^ and advances in predicting and defining antibody domains as IgG motifs that initiate and propagate compromising seed conformations, leading to aggregation.^11,12^ This extends further to the development of protective cleavable antibody fusion proteins (e.g., Anticalin-IgG fusions),^13^ as well as the site-specific re-engineering of therapeutic antibodies to counter aggregation (e.g., CC49, an anti- AG-72 mAb).^14^ Parallel needs exist for tractable and informative approaches for assaying IgG aggregation, together with approaches for preparing aggregated IgG’s as comparative controls. For the latter (and as an IgG aggregation model), transient exposure to low pH (as relevant to producer cell culture and IgG purification) is commonly and effectively employed to induce IgG aggregation^15–18^ (with heat, agitation, shear force, and high pressure as additional options). Assays of aggregated IgG’s include first those which can be essentially 2-step, and report on relative frequencies of aggregates. These include fluorescent spin dye detection, dynamic light scattering, and differential scanning calorimetry. Additional useful approaches have been reviewed with excellent detail.^19^ In addition, MALDI TOF MS that employs a high MW dynode detector is also effective for directly and quantitatively assaying antibody aggregates, including mega-Dalton IgM species.^20^

As described above, protein aggregation (including IgG’s) frequently involves conformational shifts that expose hydrophobic domains.^5,8,19^ This alters protease accessibility, and has led to the development of limited proteolysis as an aggregate assay that can additionally inform on shifts in protease accessibility within specific domains.^5,21–23^ This is to the extent that limited proteolysis (“LiP”) has been applied, together with LC-MS, to define aggregated protein profiles not only for target protein structural changes in neurodegenerative disease,^22,23^ but also for intact cell populations.^24^ For this Opinion report, one component goal is to draw attention to the suggested advantages of implementing a medium- throughput workflow for the assay of IgG aggregates that combines LiP with MALDI TOF MS.

LiP first is employed to generate peptides as hydrolyzed from aggregate- containing IgG samples, and from non-aggregated monomer IgG controls. For control samples, it’s noted that hydrophobic interaction chromatography (HIC) resins have been optimized that can effectively remove possible IgG aggregates (and yield monomeric populations)^25,26^ For the native proteolysis of IgG samples, IdeS together with Arg-C or trypsin (single- tube processing at a selected time-point and temperature) generate peptides suitable for MALDI analyses. Simultaneous (or prior) deglycosylation of IgG samples is an added compatible option (e.g., using Endo-S2). For highly aggregated IgG’s that may yield high MW peptides, samples can be processed (if necessary) via C4 resin tips (to remove possible large protein fragments prior to MALDI MS) with an option to reduce disulfide bonds prior to MALDI MS (or by the use of a reducing MALDI matrix). MALDI array multiplexing (e.g., 48-well plate) can readily be employed, and peptide m/z signatures for control IgG vs samples with suspected aggregates can be analyzed (vs positive control pH- aggregated standards) via straightforward open software such as Mass-Up [bio.tools/Mass-Up] (including principle component analyses). For antibodies with known sequences, added insight can be gained into IgG domains within aggregated IgG that may act as seed sites. Beyond rapidly providing specific quantitative peptide signatures for aggregated vs monomeric IgG antibodies, and especially for mAb’s with known sequences, this combined LiP plus MALDI MS approach promises to provide insight into antibody domains which become mis-folded to the extent of conformationally masking or revealing proteolytic sites.

## References

[1] Lu RM, Hwang YC, Liu IJ, Development of therapeutic antibodies for the treatment of diseases. J Biomed Sci. 2020;27(1):1. 31894001 10.1186/s12929-019-0592-z PMC6939334

[2] Yi SY, Wei MZ, Zhao L. Targeted immunotherapy to cancer stem cells: a novel strategy of anticancer immunotherapy. Crit Rev Oncol Hematol. 2024:104313. 10.1016/j.critrevonc.2024.104313 38428702

[3] Yagnik G, Liu Z, Rothschild KJ, Highly multiplexed immunohistochemical MALDI-MS imaging of biomarkers in tissues. J Am Soc Mass Spectrom. 2021;32(4):977–988. 33631930 10.1021/jasms.0c00473 PMC8033562

[4] Lim MJ, Yagnik G, Henkel C, MALDI HiPLEX-IHC: multiomic and multimodal imaging of targeted intact proteins in tissues. Front Chem. 2023;11:1182404. 10.3389/fchem.2023.1182404 PMC10187789 37201132

[5] Pukala TL. Mass spectrometric insights into protein aggregation. Essays Biochem. 2023;67(2):243–253. 36636963 10.1042/EBC20220103 PMC10070474

[6] Gaikwad M, Richter F, Gotz R, Site-Specific structural changes in long-term-stressed monoclonal antibody revealed with DEPC covalent-labeling and quantitative mass spectrometry. Pharmaceuticals. 2023;16(10):1418. 37895889 10.3390/ph16101418 PMC10609731

[7] Waibl F, Fernandez Quintero ML, Wedl FS, Comparison of hydrophobicity scales for predicting biophysical properties of antibodies. Front Mol Biosci. 2022;9:960194. 10.3389/fmolb.2022.960194 PMC9475378 36120542

[8] Pang KT, Yang YS, Zhang W, Understanding and controlling the molecular mechanisms of protein aggregation in mAb therapeutics. Biotechnol Adv. 2023;67:108192. 10.1016/j.biotechadv.2023.108192 37290583

[9] Zhang K, Barbieri E, LeBarre J, Peptonics: A new family of cell-protecting surfactants for the recombinant expression of therapeutic proteins in mammalian cell cultures. Biotechnol J. 2024;19(1):e2300261. 10.1002/biot.202300261 37844203

[10] Wang FAS, Fan Y, Chung WK, Evaluation of mild pH elution protein A resins for antibodies and Fc-fusion proteins. J Chromatogr A. 2024;1713:464523. 10.1016/j.chroma.2023.464523 38041974

[11] Lai PK, Fernando A, Cloutier TK, Machine learning feature selection for predicting high concentration therapeutic antibody aggregation. J Pharm Sci. 2021;110(4):1583–1591. 33346034 10.1016/j.xphs.2020.12.014

[12] Lai PK, Gallegos A, Mody N, Machine learning prediction of antibody aggregation and viscosity for high concentration formulation development of protein therapeutics. MAbs. 2022;14(1):2026208. 10.1080/19420862.2022.2026208 PMC8794240 35075980

[13] Wachter S, Angevin T, Bubna N, Application of platform process development approaches to the manufacturing of Mabcalin^™^ bispecifics. J Biotechnol. 2023;377:13–22. 37820750 10.1016/j.jbiotec.2023.10.003

[14] Lin Z, Tu B, Hemken PM, Antibody engineering to generate anti-tumor-associated glycoprotein 72 mouse recombinant CC49 IgG with improved solubility, purity, and thermal stability. J Immunol Methods. 2024;525:113606. 10.1016/j.jim.2023.113606 38145790

[15] Wu Q, Cao C, Wei S, Decreasing hydrophobicity or shielding hydrophobic areas of CH2 attenuates low pH-induced IgG4 aggregation. Front Bioeng Biotechnol. 2023;11:1257665. 10.3389/fbioe.2023.1257665 PMC10497874 37711444

[16] Saito S, Namisaki H, Hiraishi K, Engineering a human IgG2 antibody stable at low pH. Protein Sci. 2020;29(5):1186–1195. 32142185 10.1002/pro.3852 PMC7184777

[17] Lopez E, Scott NE, Wines BD, Low pH exposure during immunoglobulin G purification methods results in aggregates that avidly bind fcgamma receptors: implications for measuring Fc dependent antibody functions. Front Immunol. 2019;10:2415. 31681303 10.3389/fimmu.2019.02415 PMC6797627

[18] Imamura H, Honda S. pH-shift stress on antibodies. Methods Enzymol. 2019;622:329–345. 31155060 10.1016/bs.mie.2019.02.021

[19] Housmans JAJ, Wu G, Schymkowitz J, A guide to studying protein aggregation. FEBS J. 2023;290(3):554–583. 34862849 10.1111/febs.16312

[20] Patil AA, Liu ZX, Chiu YP, Development of a linear ion trap mass spectrometer capable of analyzing megadalton MALDI ions. Talanta. 2023;259:124555. 10.1016/j.talanta.2023.124555 37088041

[21] Grune T, Jung T, Merker K, Decreased proteolysis caused by protein aggregates, inclusion bodies, plaques, lipofuscin, ceroid, and ‘aggresomes’ during oxidative stress, aging, and disease. Int J Biochem Cell Biol. 2004;36(12):2519–2530. 15325589 10.1016/j.biocel.2004.04.020

[22] Mirbaha H, Chen D, Morazova OA, Inert and seed-competent tau monomers suggest structural origins of aggregation. Elife. 2018;7:e36584. 10.7554/eLife.36584 PMC6039173 29988016

[23] Lu H, Wang B, Liu Y, DiLeu isobaric labeling coupled with limited proteolysis mass spectrometry for high-throughput profiling of protein structural changes in Alzheimer’s disease. Anal Chem. 2023;95(26):9746–9753. 37307028 10.1021/acs.analchem.2c05731 PMC10330787

[24] Schopper S, Kahraman A, Leuenberger P, Measuring protein structural changes on a proteome-wide scale using limited proteolysis-coupled mass spectrometry. Nat Protoc. 2017;12(11):2391–2410. 29072706 10.1038/nprot.2017.100

[25] Ebert S, Fischer S. Efficient aggregate removal from impure pharmaceutical active antibodies. Bio Process International. 2011:36–42.

[26] Ewonde ER, Bottinger K, De Vos J, Selectivity and resolving power of hydrophobic interaction chromatography targeting the separation of monoclonal antibody variants. Anal Chem. 2024;96(3):1121–1128. 38190620 10.1021/acs.analchem.3c04011 PMC10809212

